# The effect of anesthetic depth on postoperative delirium in older adults: a systematic review and meta-analysis

**DOI:** 10.1186/s12877-023-04432-w

**Published:** 2023-11-06

**Authors:** Yafeng Wang, Hongyu Zhu, Feng Xu, Yuanyuan Ding, Shuai Zhao, Xiangdong Chen

**Affiliations:** 1 Department of Anesthesiology, Union Hospital, Tongji Medical College, Huazhong University of Science and Technology, Wuhan, 430022 China; 2 Institute of Anesthesia and Critical Care Medicine, Union Hospital, Tongji Medical College, Huazhong University of Science and Technology, Wuhan, 430022 China; 3 Key Laboratory of Anesthesiology and Resuscitation, Huazhong University of Science and Technology, Ministry of Education, Wuhan, China; 4 Department of Anesthesiology, Linhe District People’s Hospital, Bayannur City, 015000 China

**Keywords:** Anesthetic depth, Postoperative delirium, Older adults, General anesthesia, BIS

## Abstract

**Background:**

Postoperative delirium (POD) is an important complication for older patients and recent randomised controlled trials have showed a conflicting result of the effect of deep and light anesthesia.

**Methods:**

We included randomised controlled trials including older adults that evaluated the effect of anesthetic depth on postoperative delirium from PubMed, Embase, Web of Science and Cochrane Library. We considered deep anesthesia as observer’s assessment of the alertness/ sedation scale (OAA/S) of 0–2 or targeted bispectral (BIS) < 45 and the light anesthesia was considered OAA/S 3–5 or targeted BIS > 50. The primary outcome was incidence of POD within 7 days after surgery. And the secondary outcomes were mortality and cognitive function 3 months or more after surgery. The quality of evidence was assessed via the grading of recommendations assessment, development, and evaluation approach.

**Results:**

We included 6 studies represented 7736 patients aged 60 years and older. We observed that the deep anesthesia would not increase incidence of POD when compared with the light anesthesia when 4 related studies were pooled (OR, 1.40; 95% CI, 0.63–3.08, P = 0.41, I^2^ = 82%, low certainty). And no significant was found in mortality (OR, 1.12; 95% CI, 0.93–1.35, P = 0.23, I^2^ = 0%, high certainty) and cognitive function (OR, 1.13; 95% CI, 0.67–1.91, P = 0.64, I^2^ = 13%, high certainty) 3 months or more after surgery between deep anesthesia and light anesthesia.

**Conclusions:**

Low-quality evidence suggests that light general anesthesia was not associated with lower POD incidence than deep general anesthesia. And High-quality evidence showed that anesthetic depth did not affect the long-term mortality and cognitive function.

**Systematic review registration:**

CRD42022300829 (PROSPERO).

**Supplementary Information:**

The online version contains supplementary material available at 10.1186/s12877-023-04432-w.

## Introduction

It is well acknowledged that the older adults were more vulnerable to postoperative delirium (POD) and resulted in loss of autonomy and mortality [1]. Though the risk factors of POD were identified such as aged, ASA physical status > 2, Charlson Comorbidity Index > 2 and Mini-Mental State Examination, the drug or anesthetic interventions to reduce POD incidence remains uncertain [2].

In the past decade, electroencephalography-guided general anesthesia was considered a promising method to reduce POD incidence [3]. The essence is to avoid excessive general anesthesia intraoperatively. Evered et al. has reported that targeting light anesthesia (targeted bispectral (BIS) index 50) reduced the risk of POD [4]. However, Wildes et al. suggested EEG-guided anesthetic administration, did not decrease the incidence of POD [4, 5]. Similarly, previously meta-analysis has showed the contrary results of the outcomes of deep anesthesia on POD [6, 7]. These conflicting results mainly due to the difference in duration of excessive general anesthesia exposure and there are different definitions of light/deep anesthesia among these investigations. This limits the possibility of clinical application.

Therefore, we conducted this systematic review and meta-analysis, to provide the latest clinical evidences whether anesthetic depth was associated with the incidence of POD in older patients.

## Methods

This systematic review and meta-analysis was followed the Preferred Reporting Items for Systematic Reviews and Meta-Analyses (PRISMA) guidelines standards and the review protocol has registered with PROSPERO (Systematic review registration: CRD42022300829).

### Eligibility criteria

#### Studies

We included published articles that enrolled patients were randomly divided into light anesthesia and deep anesthesia groups via BIS or observer’s assessment of the alertness/ sedation scale (OAA/S). Eligible studies were all randomized controlled trials (RCTs), case reports and observation studies were excluded. Studies which designed to compare the difference between processed electroencephalography monitoring and usual care were excluded in our study.

#### Participants

In consideration of older adults were more vulnerable to POD, we included original studies that reported on patients aged 60 years and older and plan to undergo selective operation.

#### Interventions

It is important to unify the definitions of light and deep anesthesia. Previously study has defined BIS ranged 50–60 as light anesthesia while 30–40 as deep anesthesia [8]. In addition, Short et al. suggested BIS targeted 50 as light and BIS targeted 35 as deep anesthesia based on based on large-scale observational data and close to the first and third quartiles for mean BIS recorded in an audit of a large tertiary hospital’s anesthetic database [9]. Generally, the recommended BIS ranges are 40–60 for general anesthesia. In this light, in our study, we considered deep anesthesia as modified OAA/S of 0–2 or targeted BIS < 45 intraoperatively regardless of whether combined with spinal anesthesia. As well, the light anesthesia was considered OAA/S 3–5 or targeted BIS > 50.

#### Comparison groups

We included studies which a comparison group received light anesthesia. Correspondingly, the light anesthesia was defined as OAA/S of 3–5 or targeted BIS > 50 intraoperatively regardless of whether combined with spinal anesthesia.

#### Outcomes

The primary outcome was incidence of POD within 7days after surgery. And the secondary outcomes were mortality and cognitive function 3 months or more after surgery.

### Study selection and data extraction

#### Search strategy

A systematic search was conducted on studies published from inception to Dec 31 2021, with no restrictions on language of publication in PubMed, Embase, Web of Science and Cochrane Library by two independently researchers. Records were managed by EndNote X9.3 software to exclude duplicates. Search Strategy and keywords for these databases were described in supplementary materials.

#### Study selection and data extraction

Two independently investigators (Y.W and H.Z) screened Titles, abstracts, and full-text articles and assessed for their suitability and checked by the third researcher F.X. The disputed part was decided by F.X or by consensus. The data were extracted into a Microsoft Excel spreadsheet by one investigator (Y.W) and checked by S.Z. For the meta-analysis, collected data included: (1) first author, published year and country; (2) patient population characteristics; (3) surgery types; (4) outcome measures used; (5) the light/deep anesthesia targets and the sedation level in practice.

#### Quality assessment

We used Cochrane Risk of Bias 2.0 tool to evaluate the quality of the included studies by two investigators independently(Y.D and S.Z) [10]. Grading Recommendations Assessment, Development and Evaluation (GRADE) was used to assessed the overall certainty of evidence for primary and secondary outcomes [11]. We used the Guideline Development Tool (https://www.gradepro.org) to formulate the summary of findings table. The disputed part was solved by consensus.

### Statistical analysis

All analysis was performed using Review Manager 5.4 (The Cochrane Collaboration) software. Heterogeneity among studies was examined through Cochran Q test and the I^2^ index (I^2^ > 50% for extensive heterogeneity). If heterogeneity was low (I^2^ < 50%), the fixed-effect model (Mantel-Haenszel method) was chosen to assess combined effects; otherwise, a random-effect model (Mantel-Haenszel method) was utilized. And *P* < 0.05 was considered to indicate statistical significance.

## Results

**Fig. 1:**
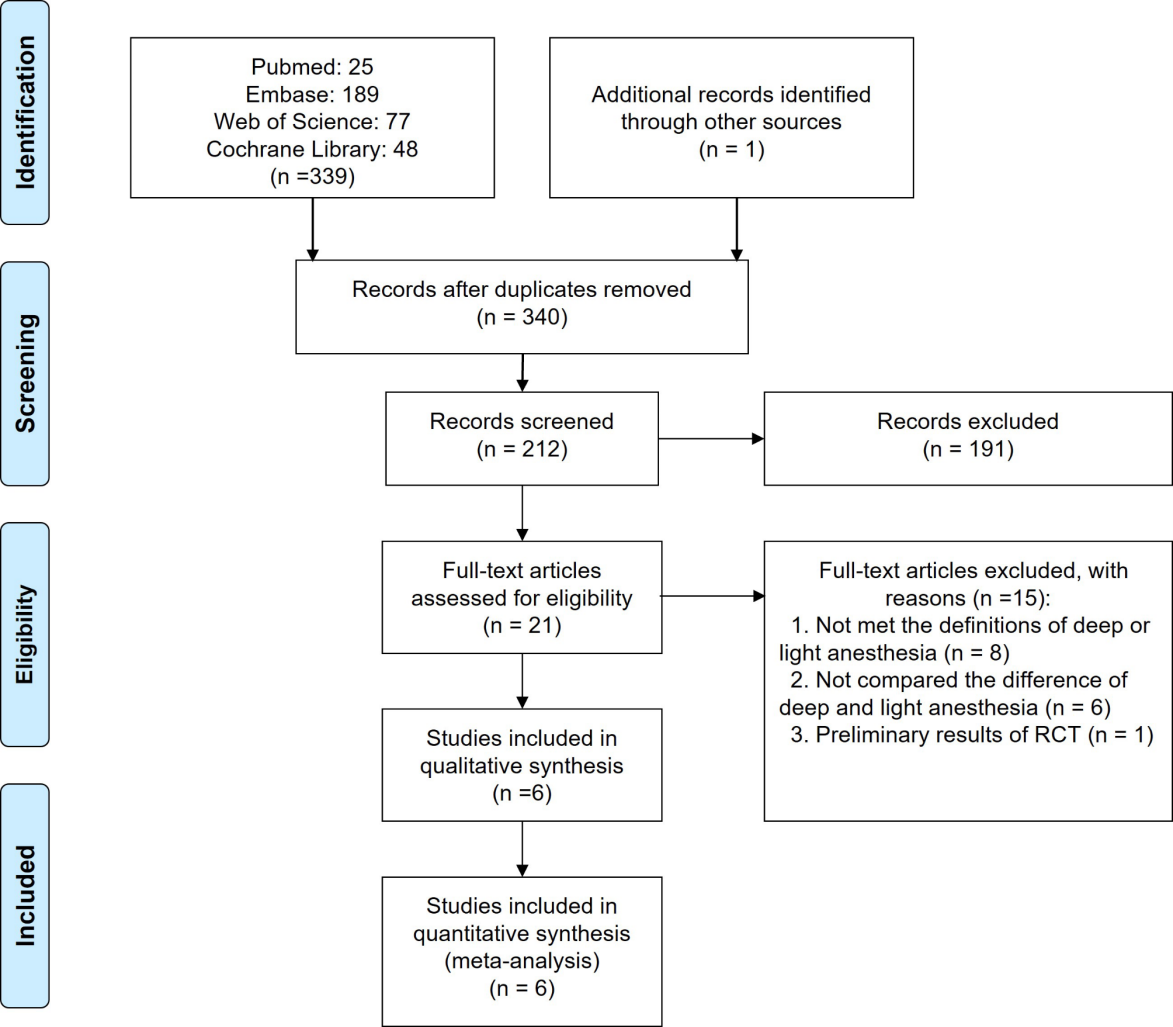
Flow diagram of study selection

We identified 339 RCTs after literature search and added 1 RCT which was identified through citation search. Subsequently, we screened 212 abstracts and identified 21 studies as requiring full-article review. Among these, 15 were excluded with reasons and 6 studies met the inclusion criteria for the review. The steps of the literature search, study selection and reasons for exclusion was shown in Fig. 1.

### Study characteristics and quality

The included 6 studies for the systematic review represented 7736 patients aged above 60 years old. All these studies were RCTs. Among these, 4 RCTs (66.7%) reported the comparation of POD incidences between light and deep anesthesia [4, 8, 12, 13] and 3 RCTs (50.0%) examined the mortality three months or more after surgery [8, 14, 15]. However, we found these studies differed significantly in type of surgery, assessment of anesthesia depth and methods for evaluating POD. Four RCTs [4, 8, 13, 14] used BIS and 2 RCTs [12, 15] used OAA/S score to evaluate the anesthesia depth. In addition, the intraoperative sedation levels were assessed in these 6 studies and actual levels of sedation were met corresponding targets (Table 1).

**Table 1:** Characteristics of Reviewed Studies

Study	Country	Surgery type	Light anesthesia					Deep anesthesia						Outcome measured	Outcome investigator
			Age	BMI	n	Target	Sedative level	Age	BMI	n		Target	Sedative level		
Evered 2021 [4]	United States	major surgery more than 2 h	70.8 (6.9)	26 (23–28)	253	BIS target 50	BIS:51 (48–53)	71.1 (6.8)	26 (22–28)		262	BIS target 35	BIS: 38 (36–40)	3D-CAM/ CAM-ICU	Trained researcher
Quan 2018 [8]	China	abdominal surgery	63.9 ± 4.56	21.4 ± 3.0	53	BIS values ranging from 45–60	84.6% of the total time BIS 45–60	65.6 ± 3.77	22.3 ± 3.4		52	BIS values ranging from 30–45	85.3% of the total time, BIS 30–45	9 neuropsychological tests	Experienced psychometrician
Short 2019 [14]	New Zealand	surgery more than 2 h	72 (7)	28 (24–32)	3316	BIS target 50	median BIS was 47.2 (IQR 43.7–50.5)	72 (7)	28 (24–32)		3328	BIS target 35	median BIS was 38.8 (36.3–42.2)	na	na
Sieber 2018a [12]	United States	hip fracture repair	81.6 (8.2)	25.2 (5.2)	100	maintained at an OAA/S score of 3–5	89.9% time of sedation fall in the desired range	82.0 (7.2)	24.8 (5.4)		100	maintained at an OAA/S score of 0–2	97.7% time of sedation fall in the desired range	Multidisciplinary consensus panel	Researcher masked to the randomization
Sieber 2018b [15]	United States	hip fracture repair	81.6 (8.2)	25.2 (5.2)	100	maintained at an OAA/S score of 3–5	89.9% time of sedation fall in the desired range	82.0 (7.2)	24.8 (5.4)		100	maintained at an OAA/S score of 0–2	97.7% time of sedation fall in the desired range	na	na
Valentin 2016 [13]	Brazil	elective non-cardiac/neurologic surgery	68.7 ± 7.7	25 ± 5	32	BIS 46–55	1th hour: 49.1 ± 11.1 2th hour: 46.2 ± 5.4 3th hour: 48.4 ± 13.5	67.2 ± 5.2	28.6 ± 5.5		40	BIS 35–45	1th hour: 38 ± 5.5 2th hour: 41.2 ± 8.1 3th hour: 44 ± 11.1	TICS and Neuropsychological Battery	na

Abbreviations: BMI: Body Mass Index; Abbreviated Mental Test score; TICS: Telephone Interview for Cognitive Status

**Fig. 2:**
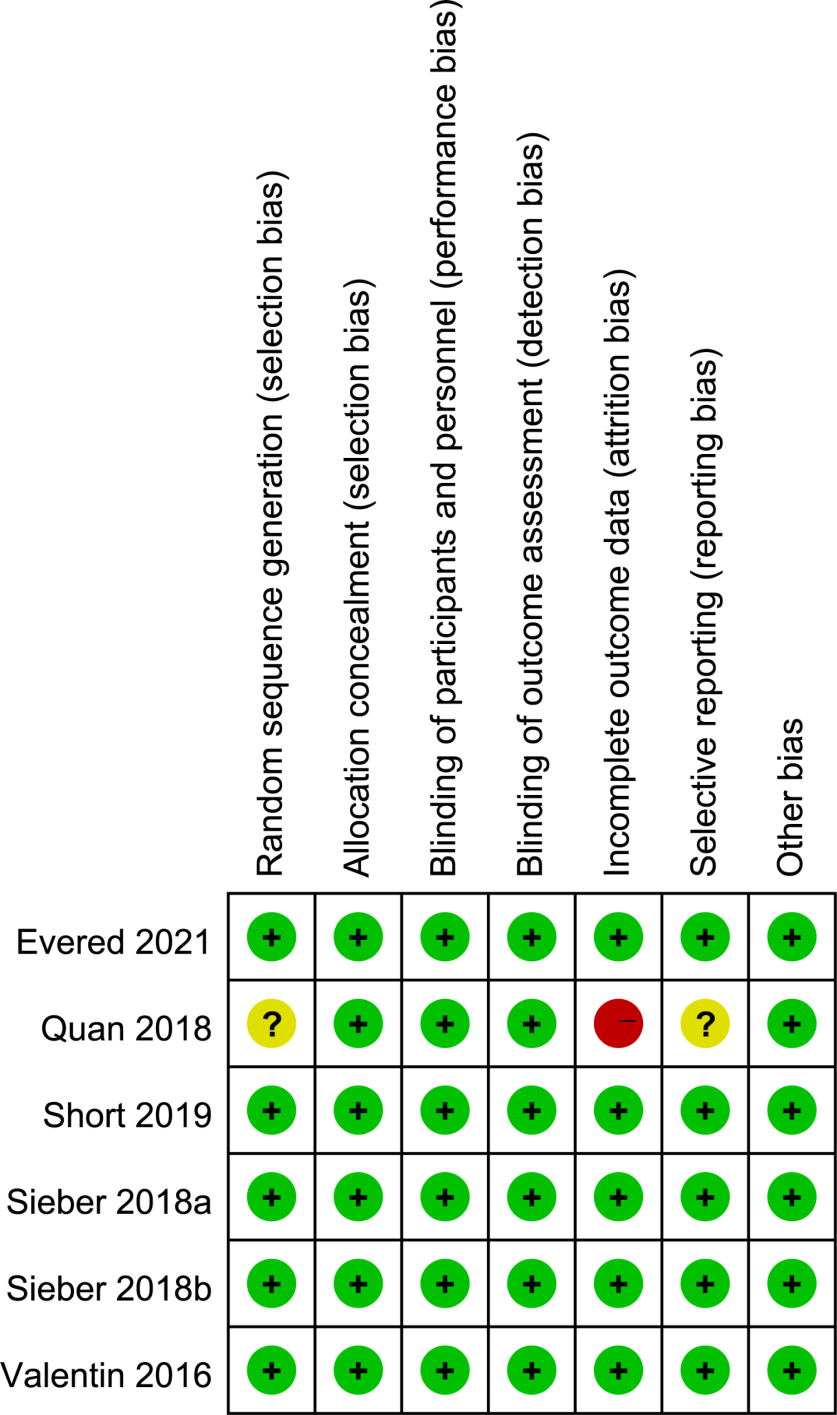
Risk of bias assessments for studies

As shown in Fig. 2, the overall risk of bias as assessed by the Cochrane was low in 5 studies [4, 12–15], the risk of selection and reporting bias was unclear in 1 study^8^, and risk of attrition bias was high in one study^8^.

### Primary outcome

**Fig. 3:**
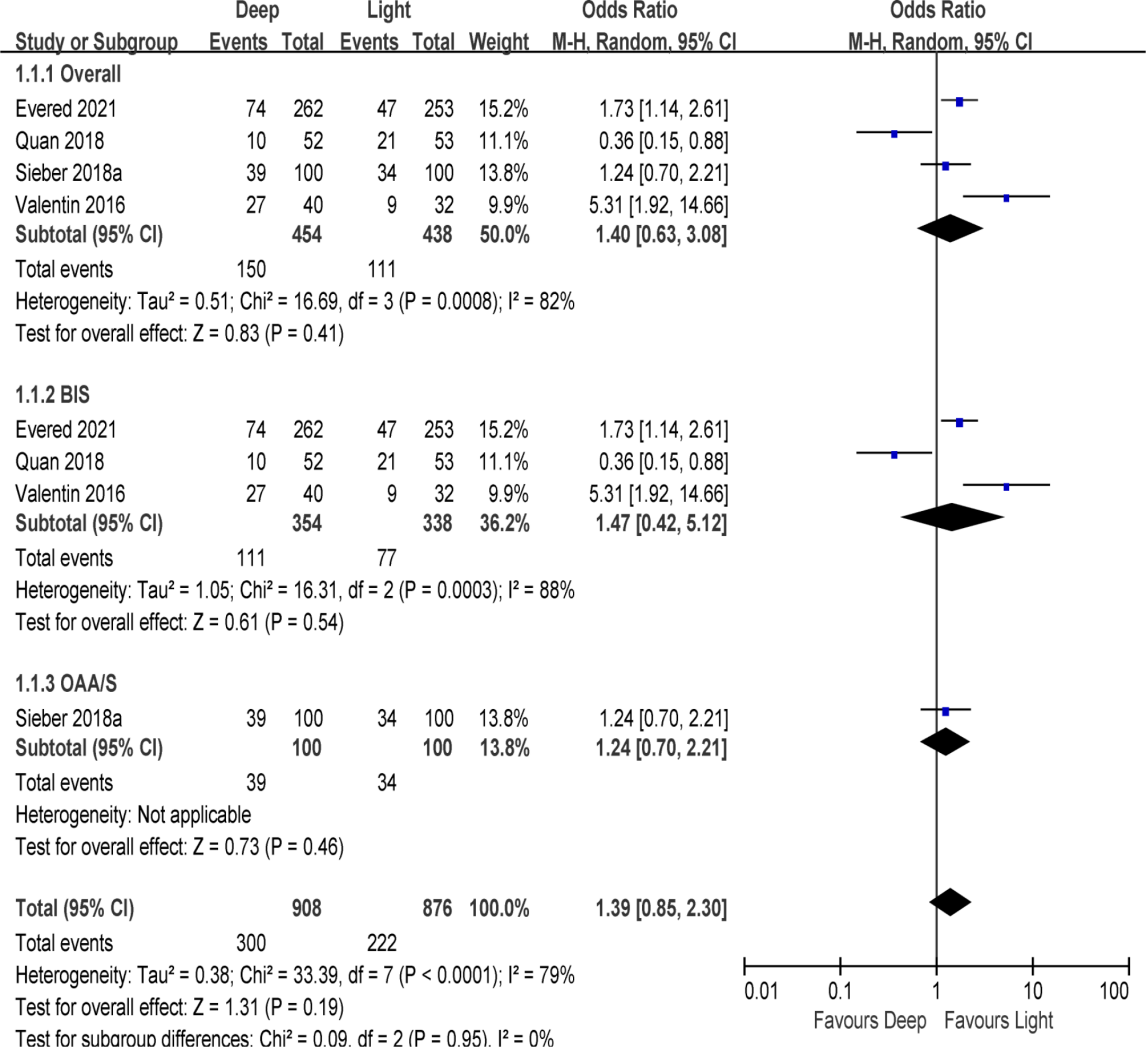
Forest plots of POD data using a random effects model

Four trails ( N = 892) compared the POD incidence in deep and light anesthesia groups [4, 8, 12, 13]. Among them, 3 studies (N = 792) [4, 8, 13] utilized targeted BIS to assess the sedative level while 1 study (N = 200) [12] used OAA/S. Meta-analysis showed that the deep anesthesia would not increase incidence of POD when compared with the light anesthesia(OR, 1.40; 95% CI, 0.63–3.08, P = 0.41, I^2^ = 82%, low certainty) (Tables 2 and Fig. 3). We subsequently performed a sensitivity analysis in order to determine whether single studies included affected the overall statistical effect. However, when omitted Quan et al. [8], the overall results showed a significant decreased incidence of POD in light anesthesia group (OR, 1.96; 95% CI, 1.05–3.65, P = 0.03, I^2^ = 66%).

**Table 2:** GRADE summary of findings

Certainty assessment							№ of patients		Effect		Certainty	Importance
№ of studies	Study design	Risk of bias	Inconsistency	Indirectness	Imprecision	Other considerations	Deep anesthesia	Light anesthesia	Relative (95% CI)	Absolute (95% CI)		
POD												
4	randomised trials	not serious	very serious^a^	not serious	not serious	none	150/454 (33.0%)	111/438 (25.3%)	**OR 1.40** (0.63 to 3.08)	**69 more per 1,000** (from 77 fewer to 258 more)	⨁⨁◯◯ Low	CRITICAL
**Cognitive function 3 months or more after surgery**												
3	randomised trials	not serious	not serious	not serious	not serious	none	35/325 (10.8%)	29/303 (9.6%)	**OR 1.13** (0.67 to 1.91)	**11 more per 1,000** (from 29 fewer to 72 more)	⨁⨁⨁⨁ High	CRITICAL
**Mortality 3 months or more after surgery**												
3	randomised trials	not serious	not serious	not serious	not serious	none	253/3480 (7.3%)	227/3469 (6.5%)	**OR 1.12** (0.93 to 1.35)	**7 more per 1,000** (from 4 fewer to 21 more)	⨁⨁⨁⨁ High	CRITICAL

**CI**: confidence interval; **OR**: odds ratio

**Explanations**

a. High I^2^ (82%) and inconsistent sensitivity analysis results which lower our certainty in effect

We next examined whether the methods of sedative assessments could influence the POD incidence. As shown in Fig. 3, there were no differences between the deep anesthesia and light anesthesia in POD incidence in studies used BIS (OR, 1.47; 95% CI, 0.42–5.12, P = 0.54, I^2^ = 88%).

Moreover, it was reported that the incidence of POD is associated with surgery types [16], due to the 4 studies draw conclusions from multiple procedures [4, 8, 12, 13], we intend to investigate the difference between major (lasting 2 h or more) and minor (lasting within 2 h) surgeries. As a result, 3 studies were included in analysis and our pooled data indicate that there were no differences between the deep anesthesia and light anesthesia in POD incidence in major surgeries (OR, 1.47; 95% CI, 0.42–5.12, P = 0.54, I2 = 88%) [4, 8, 13].

### Secondary outcomes

**Fig. 4:**
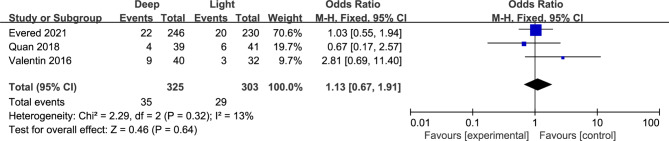
Forest plots of postoperative cognitive dysfunction 3 months after surgery data using a fixed effects model

Three studies of 628 patients reported the postoperative cognitive dysfunction incidence 3 months after surgery [3, 8, 15]. Similarly, the results showed no difference of postoperative cognitive dysfunction incidence at 3 months within deep anesthesia and light anesthesia (OR, 1.13; 95% CI, 0.67–1.91, P = 0.64, I^2^ = 13%, high certainty) (Tables 2 and Fig. 4). However, we could not identify enough studies to perform the further analysis at 12 months.

**Fig. 5:**
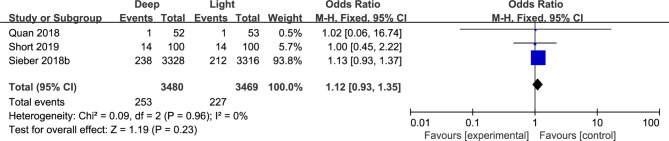
Forest plots of long-term mortality data using a fixed effects model

Three studies of 6949 patients assessed long-term mortality between deep anesthesia and light anesthesia [8, 14, 15]. It revealed no significant difference among two groups (OR, 1.12; 95% CI, 0.93–1.35, P = 0.23, I^2^ = 0%, high certainty) (Tables 2 and Fig. 5).

## Discussions

This systematic review and meta-analysis suggested that light anesthesia would not decrease the incidence of POD when compared with the deep anesthesia in older adults. And this was consistent with mortality and cognitive function 3 months or more after surgery.

POD is an important complication for older patients and the incidence of POD varies between 22% and 50% [17, 18]. Up to now, evidences on the perioperative interventions such as use of anti-psychotics and other medications, intravenous versus inhalational maintenance of anesthesia in the management of POD are still inconclusive [19, 20]. Interestingly, Luo et al. have suggested that POD may relate to the dose of anesthesia drugs used during surgery [21]. Consequently, reducing the dose of anesthetic drugs may be considerable strategies to decreased the incidence of POD for older patients. Previous meta-analysis addressing depth of anesthesia and POD have compared deep anesthesia (target BIS range 30–50) versus light anesthesia (target BIS range 46–80), and have yielded inconclusive results [6, 7]. Such as Lu et al. enrolled 4 RCTs including 340 patients and found that no significant correlation between the depth of anesthesia and POD [7]. While another meta-analysis by Li and colleagues draw a conclusion that light anesthesia was associated with a decrease in POD in comparison with deep anesthesia after evidence synthesis of 10 studies (3142 patients) [6]. This contradictory conclusion in the effect of light anesthesia on the incidence of POD mainly caused by the differences in study designs such as different duration of excessive general anesthesia exposure and targes of light/deep anesthesia. For example, the BIS value of a light anesthesia group was 49.90 ± 13.50 may similar to a deep anesthesia target in another [22, 23].

Our results suggested that light anesthesia would not decrease the incidence of POD when compared with the deep anesthesia in older adults. Major strengths of our study include controlling the range of light/deep anesthesia and identified study population. Moreover, all RCTs enrolled in analysis reported the sedation level of two groups in practice, the actual sedation levels and duration mostly conform to the range designed and differed little among these studies. Though the European Society of Anesthesiologists the American Geriatric Society, and the UK’s National Institute for Health and Care Excellence all recommend that prevent excessive anesthetic administration to patients at high risk of POD via intraoperative EEG monitoring [24–26]. Consistent with our findings, the ENGAGES trial found among older adults undergoing major surgery, EEG-guided anesthetic administration (mean to avoid excessive general anesthesia), compared with usual care, did not decrease the incidence of postoperative delirium [5]. And regional anesthesia without sedation did not significantly reduce the incidence of postoperative delirium compared with general anesthesia [27]. Interestingly, it was reported that the degree of POD may associate with EEG [28]. However, none of included studies provided the information about the severity delirium. Whether the depth of anesthesia has an effect on the degree of delirium remains undetermined. As for mortality and cognitive function 3 months or more after surgery, our pooled data showed no significant difference in light or deep general anesthesia.

Importantly, “deep anesthesia” as well as “light anesthesia” is metaphorically apt.

but quantitatively hollow [25]. So far, the clear definition of deep or light anesthesia is still lacking. The BALANCED Anesthesia Study was defined deep anesthesia as targeted BIS 35 and light anesthesia as targeted BIS 50 on the basis of previous large RCTs [14]. It is reported that the BIS targets chosen were based on large-scale observational data and were close to the first and third quartiles for mean BIS recorded in an audit of a large tertiary hospital’s anesthetic database [9, 14]. However, in our study, to include RCTs with comparable sedation intervention as possible, we chose targeted BIS < 45 as deep anesthesia and targeted BIS > 50 as light anesthesia. And we further confirmed the BIS levels were actually achieved as the target level in respective studies to ensure the reliability of our conclusions. Furthermore, the OAA/S was considered accurately reflect the clinical picture and it was reported OAA/S correlated well with BIS [29]. So that studies used OAA/S to evaluate anesthesia depth were included in the analysis.

Our study had several limitations. Firstly, only 4 RCTs in our analysis were powered to detect a difference in POD and there was significant heterogeneity of the included trials. Subsequently, we have performed several subgroup analyses and the exact reason of the observed heterogeneity was not identified. It was reported that the incidence of POD varies greatly among different types of surgery: 6%~46% in cardiac surgery [30], 5%~39% in vascular surgery [31], 8%~54% in gastrointestinal surgery [32]. And this may contribute to a high heterogeneity. Though most studies clarified their valid assessments (consensus/3D-CAM) and investigators (trained/masked researcher or experienced psychometrician), the variety of diagnostic methods undoubtedly contributed to the heterogeneity. Moreover, when we performed the sensitive analysis, the results showed an inconsistent result as the incidence of POD between light/deep anesthesia. Therefore, the results of effects of anesthetic depth on POD in older adults must be undertaken with caution and further large multicenter RCTs are needed. Secondly, as reported, deep anesthesia may increase postoperative mortality in cardiac surgery patients rather than non-cardiac surgery patients. In our study, we were failed to investigate whether the specific groups populations or surgical types were benefited from light anesthesia due to lack of corresponding literature. Thirdly, in case of the exact definitions of deep/light anesthesia are still lacking, the effect of gradients of anesthesia depth on POD should be examined.

## Conclusions

In conclusion, current data showed no difference between deep anesthesia and light anesthesia in the incidence of POD as well as mortality and cognitive function 3 months or more after surgery. However, since the total number of included RCTs was low, more multicenter RCTs with gradient anesthesia depth ranges are needed to validate this finding before clinical application in the future.

### Electronic supplementary material

Below is the link to the electronic supplementary material.

Supplementary Material 1

Supplementary Material 2

## Data Availability

The data used to support the findings of this study are included within the article.

## Abbreviations

BIS: Bispectral
OAA/S: Observer’s assessment of the alertness/sedation scale
POD: Postoperative delirium
RCTs: Randomized controlled trials
