# Supplementary material for: The effect of anesthetic depth on postoperative delirium in older adults: a systematic review and meta-analysis
Source: BMC Geriatr. 2023 Nov 6;23:719. doi: 10.1186/s12877-023-04432-w (PMC10629190; doi:10.1186/s12877-023-04432-w)
Supplement: Supplementary file 2 — Supplementary Material 2 [file 12877_2023_4432_MOESM2_ESM.doc]

**Pubmed**

#1 Search: Aged[MeSH Terms] **3382860**

#2 Search: ((((Elderly[Title/Abstract])) ) OR (Oldest Old[Title/Abstract])) OR (Older Patients[Title/Abstract]) **308796**

#3 #1 OR #2 **3466737**

#4 Search: Deep sedation[MeSH] **1451**

#5 Search: (((((Depth of Sedation[Title/Abstract]) OR (Deep Sedations[Title/Abstract])) OR (Sedations, Deep[Title/Abstract])) OR (Sedation, Deep[Title/Abstract])) OR (depth of anesthesia[Title/Abstract])) OR (deep anesthesia[Title/Abstract])  **4691**

#6 #4 OR #5  **5554**

#7 Search: (((((Bispectral Index Monitor[Title/Abstract]) OR (BIS[Title/Abstract])) OR (OAA/S[Title/Abstract])) OR (observer's assessment of awareness/sedation[Title/Abstract])) OR (bispectral index[Title/Abstract])) OR (brain function monitoring[Title/Abstract]) **97777**

#8 #6 OR #7  **102597**

#9 Search: delirium[MeSH] **11328**

#10 Search: (delirium[MeSH] AND (1000/1/1:2021/12/31[pdat])) OR ((((((((((Subacute Delirium[Title/Abstract]) OR (Delirium, Subacute[Title/Abstract])) OR (Deliriums, Subacute[Title/Abstract])) OR (Subacute Deliriums[Title/Abstract])) OR (Delirium of Mixed Origin[Title/Abstract])) OR (Mixed Origin Delirium[Title/Abstract])) OR (Mixed Origin Deliriums[Title/Abstract])) OR (Postoperative Delirium[Title/Abstract])) OR (POD[Title/Abstract])) OR (Emergence delirium[Title/Abstract]) **14336**

#11 #9 OR #10 **23892**

#12 Search: ((Randomized controlled trial[Publication Type]) OR (randomized[Title/Abstract])) OR (placebo[Title/Abstract]) **930502**

#13 #3 AND #8 AND #11 AND #12  **25**

**EMBASE**

#1 'aged'/exp 3445718

#2 'aged patient':ab,ti OR 'aged people':ab,ti OR 'aged person':ab,ti OR 'aged subject':ab,ti OR elderly:ab,ti OR 'elderly patient':ab,ti OR 'elderly people':ab,ti OR 'elderly person':ab,ti OR 'elderly subject':ab,ti OR 'senior citizen':ab,ti OR senium:ab,ti 381782

#3 #1 OR #2

#4 'anesthesia level'/exp 7281

#5 'deep sedation'/exp 3358

#6 'anesthesia depth':ab,ti OR 'anesthesia level':ab,ti OR 'anesthesia monitoring':ab,ti OR 'anesthetic depth':ab,ti OR 'anesthetic level':ab,ti OR 'anesthetic monitoring':ab,ti OR 'anesthesia depth':ab,ti OR 'anesthesia monitoring':ab,ti OR 'anesthetic depth':ab,ti OR 'anesthetic level':ab,ti OR 'anesthetic monitoring':ab,ti OR 'depth of anesthesia':ab,ti OR 'depth of anesthesia':ab,ti OR 'depth of hypnosis':ab,ti OR 'depth of sedation':ab,ti OR 'sedation depth':ab,ti OR 'sedation monitoring':ab,ti

#7 'bispectral index'/exp OR 'bis index':ab,ti 5353

#8 #4 OR #5 OR #6 OR #7 **16543**

#9 **'delirium'**/exp 36478

#10 'acute delirium':ab,ti OR 'chronic delirium':ab,ti OR 'delier; delire':ab,ti OR manifestation:ab,ti OR 'delirious state':ab,ti OR 'delirious syndrome':ab,ti OR 'delirium acutum':ab,ti 136227

#11 'pod'/exp 1047

#12 'cognition'/exp 2694285

#13 'cognitive accessibility':ab,ti OR 'cognitive balance':ab,ti OR 'cognitive dissonance':ab,ti OR 'cognitive function':ab,ti OR 'cognitive structure':ab,ti OR 'cognitive symptoms':ab,ti OR 'cognitive task':ab,ti OR 'cognitive thinking':ab,ti OR 'neurobehavioural manifestations':ab,ti OR volition:ab,ti 71779

#14 #9 OR #10 OR #11 OR #12 OR #13 2865259

#15 'randomized controlled trial'/exp 692208

#16 'controlled trial':ab,ti OR randomized:ab,ti OR 'randomised controlled study':ab,ti OR 'randomised controlled trial':ab,ti OR 'randomized controlled study':ab,ti OR 'trial, randomized controlled':ab,ti 903554

#17 #15 OR #16 1152267

#18 #3 AND #8 AND #14 AND #17 189

**Cochrane**

#1 MeSH descriptor: [Aged] explode all trees 220301

#2 (Elderly):ti,ab,kw 55563

#3 #1 OR #2 261641

#4 MeSH descriptor: [Deep Sedation] explode all trees 170

#5 (Sedation, Deep):ti,ab,kw OR (Deep Sedations):ti,ab,kw OR (Sedations, Deep):ti,ab,kw 4571

#6 #4 OR #5 4571

#7 MeSH descriptor: [Delirium] explode all trees 972

#8 (Subacute Deliriums):ti,ab,kw OR (Deliriums, Subacute):ti,ab,kw OR (Subacute Delirium):ti,ab,kw OR (Delirium, Subacute):ti,ab,kw OR (Delirium of Mixed Origin):ti,ab,kw OR (Mixed Origin Delirium):ti,ab,kw OR (Mixed Origin Deliriums):ti,ab,kw OR (POD:ti,ab,kw) 2621

#9 #7 OR #8 3509

#10 #3 OR #6 OR #9 48

**Web of Science**

#1 TS=(Aged)) OR AB=(Elderly)) OR AB=(Oldest Old)) OR AB=(Older Patients 4611111

#2 (((TS=(depth of anesthesia)) OR TS=(deep anesthesia)) OR AB=(Deep sedation)) OR AB=(Depth of Sedation)) OR AB=(Deep Sedations)) OR AB=(Sedations, Deep)) OR AB=(Sedations, Deep) **10617**

#3 ((AB=(Bispectral Index Monitor)) OR AB=(BIS)) OR AB=(OAA/S)) OR AB=(observer's assessment of awareness/sedation)) OR AB=(bispectral index)) OR AB=(brain function monitoring) **217276**

#4 #2 OR #3  **226604**

#5 ((((((TS=(delirium)) OR AB=(Subacute Delirium)) OR AB=(Delirium, Subacute)) OR AB=(Deliriums, Subacute)) OR AB=(Subacute Deliriums)) OR AB=(Delirium of Mixed Origin)) OR AB=(Mixed Origin Delirium)) OR AB=(Mixed Origin Deliriums)) OR AB=(Postoperative Delirium)) OR AB=(POD)) OR AB=(Emergence delirium)  **59018**

**#6** ((ALL=(Randomized controlled trial)) OR ALL=(randomized)) OR ALL=(placebo) **1087976**

#7 #1 AND #4 AND #5 AND #6 **77**
